# Direct nanopore sequencing of Mycobacterium tuberculosis on sputa and rescue of suboptimal results to enhance transmission surveillance

**DOI:** 10.1099/mgen.0.001709

**Published:** 2026-05-22

**Authors:** Sheri M. Saleeb, Andrea Marcos-Abellán, María Teresa Cabezas Fernández, Silvia Vallejo-Godoy, Miguel Martínez-Lirola, Guadalupe Bernal Ramírez, Marta Herranz-Martín, Sergio Buenestado-Serrano, Alfonso Pardo-Diaz, María Jesús Ruiz Serrano, Patricia Muñoz, Laura Pérez-Lago, Darío García de Viedma

**Affiliations:** 1 Department of Clinical Microbiology and Infectious Diseases, Gregorio Marañón General University Hospital, Gregorio Marañón Health Research Institute (IiSGM), Madrid, Spain; 2 CIBER of Enfermedades Respiratorias (CIBERES), Madrid, Spain; 3 Doctoral School, Autonomous University of Madrid, Madrid, Spain; 4 Microbiology Department, Torrecárdenas University Hospital Complex, Almería, Spain; 5 Department of Preventive Medicine, Public Health and Epidemiological Surveillance, Poniente University Hospital, Almería, Spain; 6 Department of Technology, Extremadura Center for Advanced Technologies (CETA-CIEMAT), Trujillo, Spain; 7 Department of Medicine, Complutense University of Madrid, Madrid, Spain

**Keywords:** direct on sputa, genomic epidemiology, nanopore sequencing, transmission, tuberculosis

## Abstract

Whole-genome sequencing (WGS) has enhanced precision to predict antimicrobial resistance and track transmission in *Mycobacterium tuberculosis* (MTB). Due to the slow-growing nature of MTB, genomic results are unavoidably delayed; however, few efforts have been made to accelerate them by performing WGS directly on respiratory specimens. Most culture-free efforts in the literature have focused on the acceleration of resistance prediction. The present study provides further evidence to the only preceding study aiming to accelerate the precise delineation of transmission, coupling culture-free WGS to a surveillance programme. Our study is distinguished from its predecessor by being the first to apply flexible nanopore sequencing for the purpose of further accelerating the process. A total of 71 sputa were selected for the study, in which we only applied a procedure to deplete human DNA, thus avoiding other costly and cumbersome alternatives based on capture baits. Optimal results (>90% of genome covered, mean coverage >45× and >70% genome covered >20×) were obtained from 33.8% of the cases, which allowed the corresponding cases to be ruled in or out of transmission clusters, close to their diagnosis. A further 12.6% of the samples yielded suboptimal results (15.5%–90.92% at >10×), which could also be exploited thanks to our rescue pipeline; the rationale was to attempt to identify, from the suboptimal sequences, the informative SNPs that are markers for relevant transmission clusters in our population. The rescue pipeline facilitated pre-allocation of new cases to pre-existing clusters and moreover enabled the specification of genomic relationships between new cases and preceding ones in the cluster. In summary, the present study has demonstrated that epidemiologically valuable information can be obtained directly from sputum in approximately half of the samples analysed. This study represents a new advancement in the pursuit of enhancing faster comparative genomics, at diagnosis, for epidemiological applications.

**Impact Statement**

This study provides new data to the scarce literature aiming to perform genomic analysis on tuberculosis directly on clinical specimens, without the need to wait until the culture of this slow-growing microorganism is available. Most of the limited efforts to implement genomic analysis on sputa are targeted approaches, devoted to *Mycobacterium tuberculosis* (MTB) identification, resistance mutations or general phylogenetic analysis. Genomic epidemiology requires the analysis of the complete genome, beyond the genetic targets relevant for diagnosis, and the studies aiming it directly on respiratory specimens require either demanding long procedures or enrichment by capturing MTB material. We based our analysis on a simpler procedure, only pursuing to deplete human DNA, thus avoiding other costly and cumbersome alternatives based on capture baits. Our study is distinguished from its predecessors by being the first to apply flexible case-by-case nanopore sequencing for the purpose of further accelerating the process, coupling culture-free WGS to a surveillance programme to accelerate the precise delineation of transmission. In addition to the 33.8% of the cases in which we obtained sequencing coverages and depth as optimal as those obtained from culture, we developed a strategy, based on identifying cluster-specific marker SNPs, which allowed us to exploit a further 12.6% of the samples which had yielded suboptimal results. This study represents a new advancement in the pursuit of enhancing faster comparative genomics, at diagnosis, for epidemiological applications.

## Data Availability

The sequences generated for Nanopore were deposited in the ENA (project number PRJEB97396). Human reads have been removed from the sequences to maintain the anonymity of the sequence.

## Introduction

Whole-genome sequencing (WGS) has significantly enhanced the precision with which we can track *Mycobacterium tuberculosis* (MTB) transmission, thereby optimizing the responses required for transmission control and guiding epidemiological interventions [1]. However, the implementation of these interventions necessitates the minimization of the time interval between the diagnosis of each new case and the availability of genomic epidemiology analyses. The majority of genomic analysis conducted on MTB for epidemiological purposes is performed on cultures, a process that introduces significant delays due to the slow growth rate of MTB [2]. Consequently, the optimal approach to expedite response times in genomic epidemiology entails a direct transition from cultures to sequencing on clinical specimens, thereby circumventing the culture step entirely to facilitate enhanced epidemiological decision-making. Despite the endeavours to perform WGS directly on sputa [3,6], these are still limited, with several requiring either demanding long procedures [4] or enrichment by capturing MTB material [2,3, 5]. All of the aforementioned aspects indicate that WGS analysis on sputa remains challenging [7]. Only targeted approaches, devoted to MTB identification, resistance mutations or general phylogenetic analysis, have been demonstrated to be successful [8,11].

In the present study, the performance of fast nanopore sequencing was evaluated directly on sputa, without the need for additional capture/enrichment procedures, within the context of our long-term genomic epidemiology surveillance/intervention programme in Almeria [12]. This approach was adopted to ensure a faster analysis, with the objective of tailoring epidemiological interventions. Furthermore, an alternative rescue pipeline was developed to exploit suboptimal sequences and assign them to pre-existing relevant clusters by focusing on cluster marker SNPs. The present study constitutes a further progression in the direction of real-time genomic surveillance in routine laboratory settings.

## Methods

### Sample collection

Clinical samples (decontaminated sputa with a bacillary load ranging, according to smear grade, from +1 to +4) were routinely collected at Torrecárdenas University Hospital (Almeria, Spain). The samples were decontaminated using a solution of *N*-acetyl-l-cysteine and NaOH (final NaOH concentration: 1%), followed by pH neutralization with 10–15 ml of phosphate buffer (pH 6.8) for resuspension. The samples were then transferred frozen at −20 °C to our laboratory at Gregorio Marañón Hospital (Madrid, Spain).

### DNA purification

Ten to 15 ml of decontaminated sputa was subjected to a centrifugal process at 8,500 r.p.m. for 40 min at a temperature of 4 °C. Thereafter, the supernatant was discarded, and the resultant pellet was suspended in 500 µl of host cell lysis (AHL) buffer. The depletion of human DNA, extraction and purification were performed by the QIAamp DNA Microbiome Kit (Qiagen, Hilden, Germany), commonly used to deplete human DNA in the context of microbiome analysis [13,15], following the manufacturer’s instructions. The final concentration and washing of DNA was achieved by employing 1.8X magnetic beads (CleanNGS, GC biotech, Netherlands) and eluting in 12 µl nuclease-free water. The quantification of the purified DNA was conducted using a Quantus fluorometer.

### Real-time PCR

In order to ascertain the relative proportion of human, bacterial and MTB complex (MTBC) DNA in the purified DNA samples, three real-time PCRs were performed using TaqMan probes to target, respectively, *RNase P*, *16S rRNA* and *Rv2341* [16], using the following primers/probes: *RNase P*: forward-AGATTTGGACCTGCGAGCG, reverse-GAGCGGCTGTCTCCACAAGT, Probe-HEX-TTCTGACCTGAAGGCTCTGCGCGBHQ-1; *16S rRNA*: forward 5′-TGGAGCATGTGGTTTAATTCGA-3′, reverse 5′-TGCGGGACTTAACCCAACA-3′, probe 5′-HEX-CACGAGCTGACGACARCCATGCA-3′; and for *Rv2341* forward 5′-GGCCGCTCATGCTCCTTGGAT-3′, reverse 5′-AGGTCGGTTCGCTGGTCTTG-3′ and probe 5′-6-FAM-TGAGTGCCTGCGGCCGCAGCGC-BHQ-1-3′. The PCR reactions included 0.5 μM of each RNase P primer and 0.25 μM of its probe; 0.9 μM of each 16S rRNA primer and 0.4 μM of its probe; and 0.25 μM of each Rv2341 primer with 0.35 μM of its probe, with 2 µl of the LightCycler FastStart DNA Master HybProbe (Roche, USA) buffer, containing 3.2 mM Mg2+, in a final reaction volume of 20 µl. The PCR conditions were for *RNase P* 55 °C for 5 min, 95 °C for 5 min, followed by 35 cycles of 95 °C for 15 s and at 60 °C for 30 s, concluding with 40 °C for 30 s; for *16S rRNA*, 95 °C for 10 min, 40 cycles of 95 °C for 10 s, 60 °C for 20 s, 72 °C for 1 s and finally 40 °C for 30 s; and for *Rv4321*, 95 °C for 10 min, 55 cycles for 95 °C for 10 s, 60 °C for 20 s, 62 °C for 1 min and, finally, 40 °C in 30 s.

### WGS

Libraries were prepared from all the purified DNA obtained from each sputum, using the Rapid PCR Barcoding kit (SQK-RPB004 and SQK-RPB114) as per the manufacturer’s instructions with modifications in increasing the PCR cycles to 25 cycles (Oxford Nanopore Technologies, Oxford, UK). A total of 5% DMSO (Dimethyl sulfoxide) was added to improve DNA denaturation during the amplification step. As recommended in the first step of the library preparation protocol (fragmentation step), 5 ng of DNA should be adjusted in 3 µl. However, since the amount of DNA obtained from sputa was below these values, 2–3 libraries were prepared per specimen, to be later pooled. The libraries were loaded and run on MinION flow cells (R.9.4.1 FLO-MIN106 and R10.4.1 FLOW-MIN114). To calculate the molar concentration which was loaded per sample, we used the mean length for the sequences obtained in our nanopore runs (3,300 bp).

Genome coverage and depth were monitored during the sequencing run using RAMPART v1.2.0. The sequencing run was permitted to continue until achieving a minimum of 70% genome coverage at 20× depth in all the sequences, or until 72 h had elapsed, whichever occurred first. Furthermore, when the progression of the run indicated that the required coverage thresholds were not going to be obtained for some of the samples, the run was aborted to allow the flow-cell to retain sufficient pores for reuse in other projects.

### Bioinformatic analysis

#### Standard pipeline

The subsequent analysis of the data was conducted using an in-house pipeline (*https://github.com/MG-IiSGM/prokaION*) [17]. Initially, the raw fast5/pod5 files underwent a preprocessing step, whereby they were converted into fastq format through base-calling and barcoding using Guppy v6.4.6, or Dorado v0.9.6. Subsequently, the quality of the data was assessed using NanoFilt v2.8.0 and NanoPlot v1.41.0 in order to ensure the reliability and informativeness of the data for subsequent analyses. The resulting reads were then mapped using a hypothetical MTB ancestral genome [18] as a reference, with minimap2 v2.28 employed for this purpose. Subsequently, SNPs were identified with FreeBayes v1.3.2. The detected variants were subsequently annotated with SnpEff v5.1, and occasional low-coverage positions (>12×) were recalibrated using joint variant calling. The identification of human and bacterial samples was conducted utilizing the Kraken2 2.1.3 and Mash v2.3 software.

For the assignment of new cases to clusters, we introduced the new sequences into the local phylogeny obtained for the 1,173 cases sequenced in Almería. In instances where a new sequence was found to be in close proximity to a pre-existing sequence, either within the same clade or as a sister taxon originating from the same rooted branch, all related sequences were processed to generate a pairwise SNP distance matrix. A threshold of 12 SNP differences was applied: sequences within this limit were considered part of the same cluster, whereas sequences exceeding this threshold were classified as outgroup ‘orphans’. Clusters were further refined and considered definitive when a pairwise distance of fewer than five SNPs was confirmed by visual inspection.

The alignment and SNPs were then visualized and inspected using the IGV (Integrative Genomics Viewer) program. Median-joining networks of genome-related isolates were constructed from the SNP matrix generated by PopART software [19].

### Rescue pipeline to exploit suboptimal sequences

Firstly, the identification of a set of marker SNPs for a selection of relevant clusters (those growing in the 2021–25 period) was undertaken to ascertain the capacity of these informative marker SNPs to be called from suboptimal sequences. The process involved, firstly, the identification of common SNPs shared by all the representatives of the relevant clusters that had been selected. Then, cluster marker SNPs were identified by filtering cluster common SNPs against an in-house global reference database comprising SNPs from 109,648 high-quality sequences from publicly available datasets, representing the geographic and phylogenetic diversity of MTB lineages 1–9. The inclusion of sequences was contingent upon the fulfilment of stringent selection criteria, encompassing the following criteria: only datasets obtained by Illumina, the presence of complete metadata, exclusive taxonomic assignment to MTB and the attainment of specified quality thresholds (>70% of the genome covered at >20×depth and <25% of the genome uncovered). The global SNP database contains a total of 2,042,025 polymorphic positions (allele depth >20, allele frequency >70%). The final set of marker SNPs for the clusters of interest was subsequently utilized in a rescue pipeline designed to exploit suboptimal sequences. In instances where more than one marker SNP is found within the same gene, they are not taken into consideration, as it is deemed to be an extremely improbable occurrence in MTB and it may suggest false calls.

Suboptimal sequences were preassigned as likely to correspond to one of the relevant clusters, if by applying the rescue pipeline, >25% of the marker SNPs of one of the relevant clusters were called at ≥5×. The cases who were preassigned into pre-existing clusters by the rescue pipeline from suboptimal sequences obtained from sputa were confirmed from high-quality sequences obtained later from WGS of the corresponding cultures inoculated by the same sputa.

### Epidemiological data collection

The Almeria TB Prevention and Control Program (TBPCP) obtained and analysed epidemiological data. The TBPCP involves epidemiologists, microbiologists, professionals in preventive medicine units, nurses, social workers, professionals in primary care centres and three hospital tuberculosis clinical units. The TBPCP is integrated into the Epidemiological Surveillance System of Andalusia (SVEA), an extensive surveillance system integrated within the Spanish Epidemiological Surveillance Network (RENAVE). Community health workers belonging to local non-governmental organizations (Red Cross and the Consortium of Entities for Comprehensive Action with Migrants) operating within the designated territory were involved, providing support to the public health system and serving as cultural mediators and translators.

A data collection form was used to record the main cluster characteristics, transmission environments and risk factors identified. To supplement the findings, a comprehensive review of the patients’ clinical records and additional interviews were conducted for the cases in clusters that required a more profound investigation.

## Results

### General performance of nanopore sequencing from sputa

The analysis encompassed 71 stain-positive sputa from different patients. Forty-one of these corresponded to prospective non-selected consecutive cases, diagnosed in the period January–October 2024. The remaining cases were analysed retrospectively, in two different periods, including sputa corresponding to the years 2007–2015 and November 2024–June 2025, respectively (Table 1). A total of 25 nanopore sequencing runs were conducted, incorporating libraries for 1–5 specimens per run (median 3 samples; 2.2–3.27 fmol/sample).

**Table 1.** General performance of the 25 sequencing runs for the 71 sputa analysed

Run no.	Patient ID	Sample no.	Bacillary load	Year of collection (prospective/retrospective)		Run time	Mean coverage	Performance category	Ratio MTB reads/human reads	ONT chemistry	Total no. of reads
R1	33490676-2	1	4+	2023	Prospective	22 h 13 min	2.15	Poor	3.43	R09	679.85 k
	3255	2	4+	2024	Prospective		21.95	Suboptimal	7.23		
	3257	3	2+	2024	Prospective		0.74	Poor	0		
R2*	3268	4	4+	2024	Prospective	2 h 44 min	0.02	Poor	0.95	R10	34.32 k
	3262	5	4+	2024	Prospective		0.03	Poor	0.74		
	3265	6	4+	2024	Prospective		0.01	Poor	0.17		
R3	3281	7	4+	2024	Prospective	26 h 44 min	0.16	Poor	0.12	R10	102.62 k
	3290	8	3+	2024	Prospective		1.04	Poor	2.86		
R4	1597	9	3+	2012	Retrospective	3 h 14 min	1.92	Poor	2.57	R09	78.21 k
R5	1165	10	4+	2009	Retrospective	24 h 27 min	825.29	Optimal	74	R09	2.8 M
	3278	11	4+	2024	Prospective		29.2	Suboptimal	0.04		
	3280	12	4+	2024	Prospective		3.88	Poor	0.18		
R6	691	13	3+	2007	Retrospective	4 h 17 min	69.9	Optimal	37.29	R09	611.42 k
R7	1598	14	4+	2012	Retrospective	49 h 20 min	10.7	Suboptimal	1.02	R09	4.34 M
	755	15	3+	2007	Retrospective		15.53	Suboptimal	0.01		
	3299	16	4+	2024	Prospective		489.2	Optimal	34.46		
R8	3313	17	4+	2024	Prospective	24 h 50 min	1.56	Poor	0.36	R10	1.17 M
	3315	18	4+	2024	Prospective		5.76	Suboptimal	2.03		
	3296	19	4+	2024	Prospective		816.69	Optimal	99.84		
R9	3283	20	4+	2024	Prospective	28 h 31 min	128.29	Optimal	1.36	R10	2.93 M
	3292	21	2+	2024	Prospective		0.07	Poor	0.38		
	630	22	3+	2007	Retrospective		0.7	Poor	1.5		
	3252	23	4+	2024	Prospective		8.37	Suboptimal	0.36		
R10	3302	24	4+	2024	Prospective	25 h 10 min	144.05	Optimal	4.26	R10	2.75 M
	3319	25	4+	2024	Prospective		1,141.39	Optimal	58.36		
	3295	26	4+	2024	Prospective		70.64	Optimal	0.23		
R11	3309	27	3+	2024	Prospective	29 h 22 min	99.05	Optimal	0.44	R10	4.09 M
	3325	28	4+	2024	Prospective		122.07	Optimal	49.7		
R12	3324	29	2+	2024	Prospective	72 h	3.46	Poor	0.02	R10	4.09 M
	3330	30	4+	2024	Prospective		943.65	Optimal	22.86		
	3333	31	3+	2024	Prospective		124.38	Optimal	0.34		
	3334	32	4+	2024	Prospective		221.76	Optimal	7.06		
R13	3344	33	2+	2024	Prospective	69 h 59 min	127.68	Optimal	187.8	R10	8.16 M
	3345	34	4+	2024	Prospective		2,863.31	Optimal	107		
	3339-33272407	35	1+	2024	Prospective		6.21	Poor	0.01		
	3339-14216402	36	1+	2024	Prospective		0.02	Poor	0		
R14	3364	37	4+	2024	Prospective	4 h 12 min	166.21	Optimal	28.52	R10	250.77 k
	3366	38	3+	2024	Prospective		0.02	Poor	0.46		
	3367	39	4+	2024	Prospective		0.24	Poor	0.5		
R15	3370	40	2+	2024	Prospective	25 h 19 min	4.1	Poor	0.01	R10	2.54 M
R16	1510	41	4+	2011	Retrospective	72 h	5.68	Suboptimal	0	R10	581.17 k
	1876	42	4+	2014	Retrospective		18.78	Suboptimal	0.13		
R17	1925	43	3+	2014	Retrospective	45 h 24 min	0.83	Poor	0.01	R10	881.53 k
	2024	44	4+	2015	Retrospective		1.48	Poor	0.06		
R18	3373	45	3+	2024	Prospective	17 h 53 min	0.33	Poor	0.39	R10	47.87 k
	3378	46	4+	2024	Prospective		2.02	Poor	24.88		
	3379	47	3+	2024	Prospective		0.12	Poor	7.12		
	3382	48	3+	2024	Prospective		0.18	Poor	0.06		
R19	3388	49	1+	2024	Prospective	25 h 57 min	0.43	Poor	0.2	R10	374.76 k
R20	3389	50	4+	2024	Prospective	n/a	4.17	Poor	0.59	R10	52.68 k
	3393	51	4+	2024	Prospective		0.44	Poor	0		
R21	3417	52	2+	2025	Retrospective	47 h 1 min	1.37	Poor	115.69	R10	3.2 M
	3420	53	1+	2025	Retrospective		9.76	Poor	1.37		
	3429	54	4+	2025	Retrospective		56.57	Optimal	8.24		
	3419	55	2+	2025	Retrospective		9.76	Poor	0.82		
R22	3450	56	4+	2025	Retrospective	71 h 34 min	224.37	Optimal	1.97	R10	4.625 M
	3455	57	1+	2025	Retrospective		10.93	Poor	0		
	3465	58	4+	2025	Retrospective		100.6	Optimal	5.07		
	3467	59	2+	2025	Retrospective		1	Poor	0.41		
R23	3474	60	3+	2025	Retrospective	28 h 1 min	4.32	Poor	8.17	R10	1.55 M
	3479	61	4+	2025	Retrospective		0.07	Poor	0		
	3481	62	4+	2025	Retrospective		1.77	Poor	12.85		
	3472	63	4+	2025	Retrospective		45.54	Optimal	3.01		
R24	3462	64	3+	2025	Retrospective	72 h	131.68	Optimal	29.88	R10	5.62 M
	3488	65	4+	2025	Retrospective		978.95	Optimal	130.63		
	3500	66	1+	2025	Retrospective		3.76	Poor	3.27		
	3502	67	4+	2025	Retrospective		0.01	Poor	0.24		
R25	3503	68	4+	2025	Retrospective	53 h 2 min	97.66	Optimal	294.04	R10	3.92 M
	3524	69	1+	2025	Retrospective		0.29	Poor	4.06		
	3523	70	4+	2025	Retrospective		102.2	Optimal	30.57		
	3522	71	3+	2025	Retrospective		64.61	Suboptimal	0.21		

Bacillary loads correspond to: 1+: <1/field, 2+: 1-9/field, 3+: 10-100/field, 4+:>100/field).

*Run including two additional samples not corresponding to this study.

**Fig. 1.**
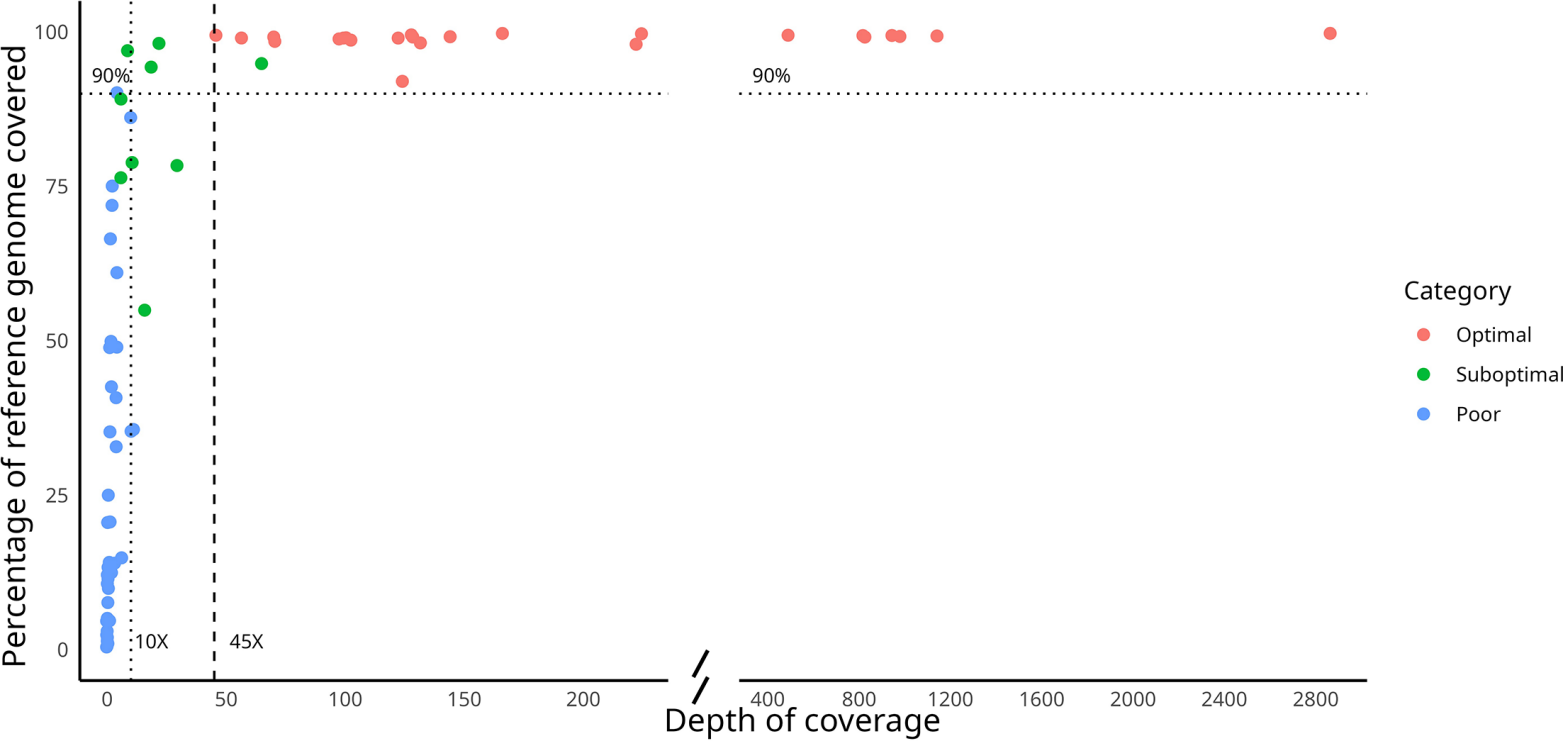
Sequencing performance (percentage of the MTB reference genome that is covered and mean depth of coverage), for the specimens in the study that have been categorized as optimal, suboptimal or poor. Each dot in the graph corresponds to one sputum sample. X-axis is truncated to avoid the empty spaces corresponding to the intermediate values along the X-axis (values between 225 and 400) which were not obtained from any of the specimens assayed.

Among the study samples, 24 of them (33.8%) led to optimal results (>90% of genome covered, mean coverage >45× and >70% genome covered >20×; Fig. 1); the shortest run that allowed us to achieve >90% at mean coverage depth >50× in a sample took 4 h and 12 min (Table 1). Another nine samples (12.6%) yielded suboptimal results, with a range of 15.5%–90.92% genome covered at >10×. The remaining 38 sputa (53.5%) were classified as poor results, with genome covered at ≤15% at >10× (see Table S1, available in the online Supplementary Material).

**Fig. 2.**
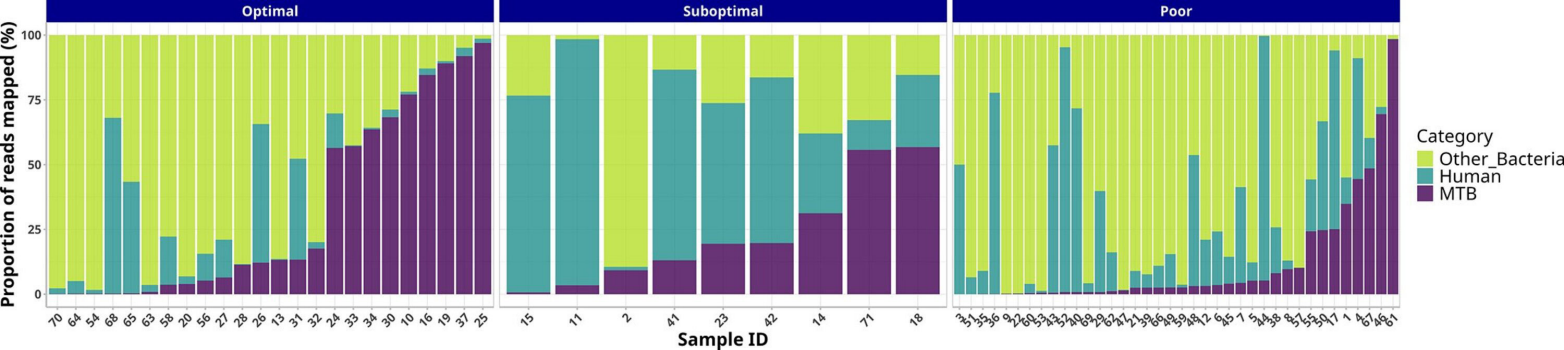
Proportion of reads mapping with MTB, human or bacteria from the sequences obtained in each sputum sample for the optimal, suboptimal and poor sequencing categories.

**Fig. 3.**
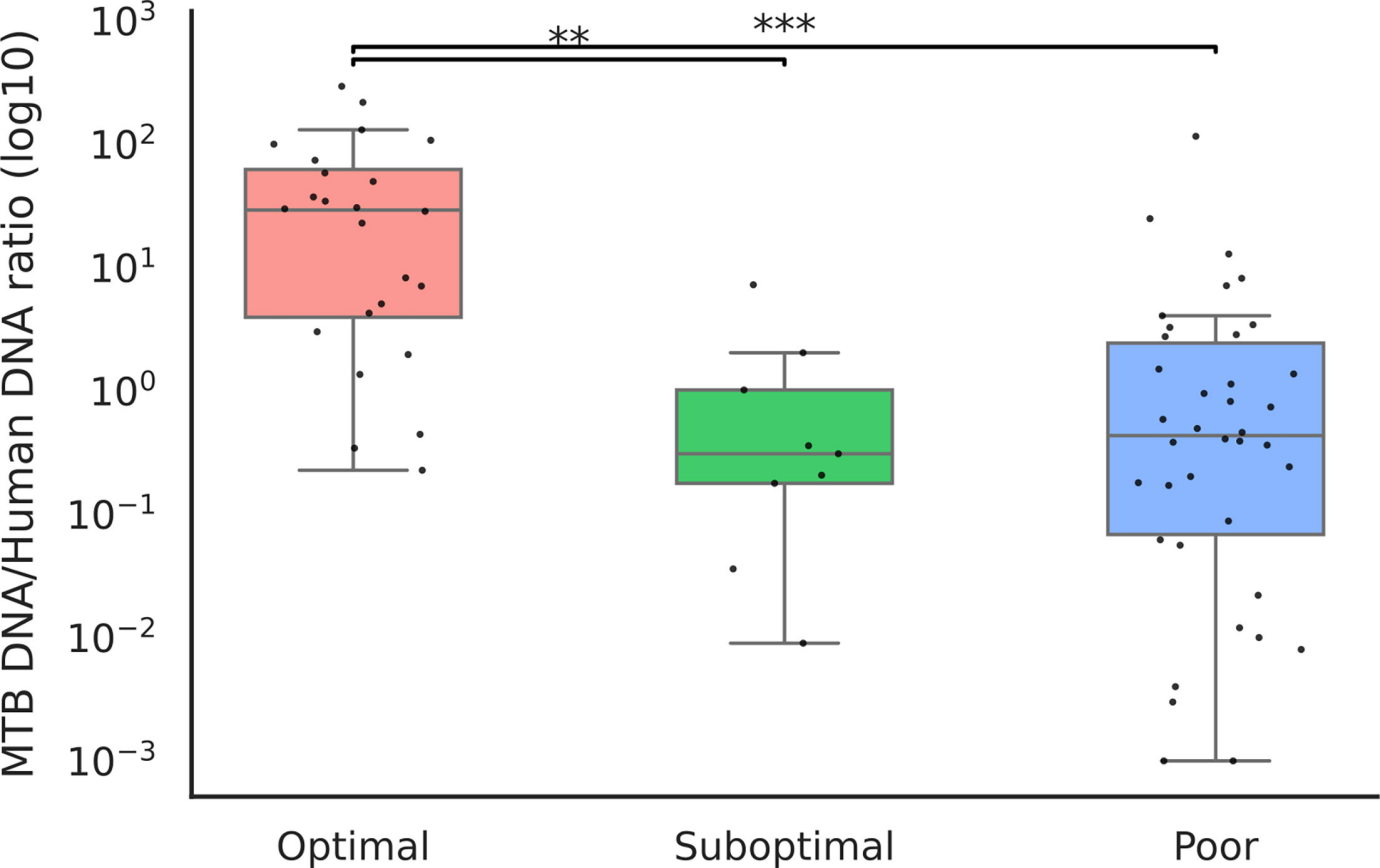
MTB DNA/human DNA ratio distribution for the samples leading to optimal, suboptimal and poor sequencing performances. **P<0.01 and ***P<0.001.

For each category (optimal, suboptimal and poor), the percentage of reads corresponding to human, bacteria other than MTB and MTB was determined (Fig. 2). Despite incorporating a procedure that depletes human DNA in the extraction stage, a substantial variation in efficiency was observed for the depletion. A direct correlation between the proportion of MTB reads and the sequencing performance was not found, as (i) optimal results were obtained even in samples containing a majority proportion of interfering DNA from either other bacteria or human (Fig. 2) and (ii) poor results were obtained even in samples with a majority of MTB. When we obtained the ratio of MTB DNA to host DNA for each sample (Table 1), the higher values (>45.5) corresponded to an optimal sequencing performance and the lowest values (<10.93) to a poor performance. Moreover, an association was found between this ratio and the optimal or suboptimal/poor sequencing performance (Fig. 3; Kruskal–Wallis chi-square=25.9867, df=1, *P*-value=2.2755e-06).

### Evaluation of factors that might predict sequencing performance

**Fig. 4.**
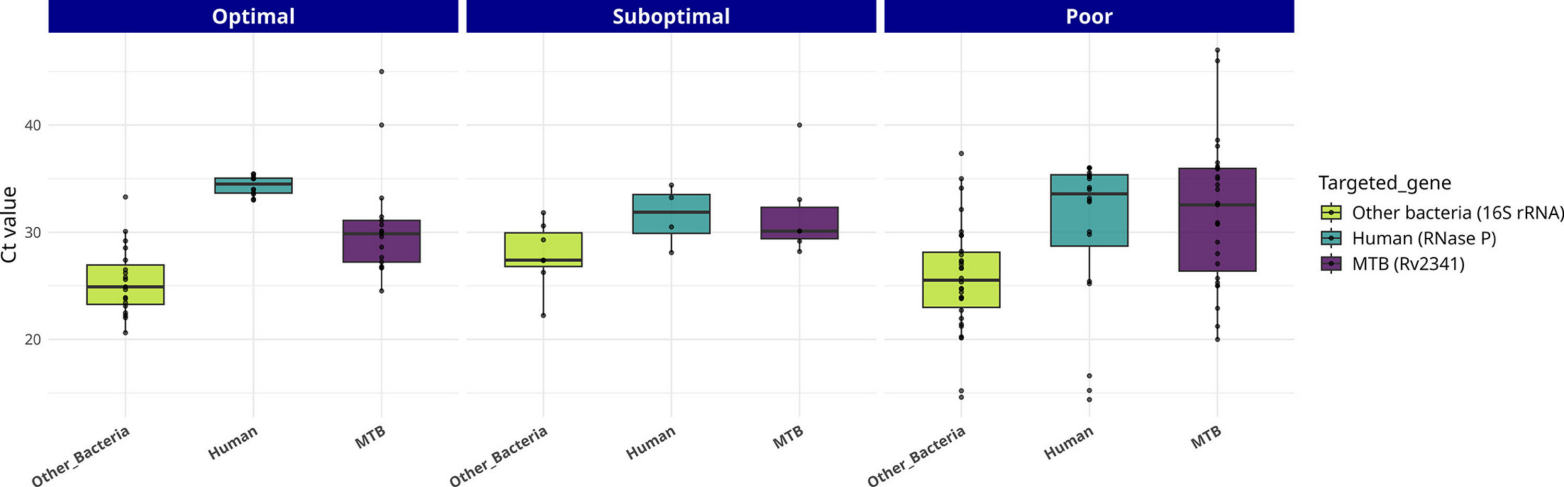
Ct values for real-time PCRs (RT-PCRs) targeting human (RNase P), MTB (Rv2341) and other bacteria (16S rRNA) for the optimal, suboptimal and poor sequencing categories. Boxplots represent the median (central line) and interquartile range (IQR; box), with whiskers extending to 1.5× IQR. Individual points correspond to single samples.

Our aim was to determine whether certain factors could be utilized to predict when sequencing from sputa is likely to yield optimal, suboptimal or poor results. With regard to the bacillary load, as determined by smear grade, the majority of the samples (76%) were classified as 3+/4+ specimens (Table 1). This precluded an assessment of the potential association between bacterial load and sequencing performance. However, it was observed that in all but one of the cases with low bacillary load, poor results were obtained, whilst all the optimal results corresponded to higher bacillary loads (Table 1). It is also true that poor results were obtained from sputa with high bacillary loads, indicating that smear grade by itself does not predict the quality of the results. Therefore, we aimed to estimate, in addition to the presence of MTB in the samples, the presence of interfering DNA. This was achieved by examining the Ct values for three real-time PCRs targeting bacteria, human DNA and MTB (Fig. 4).

As was previously observed, the efficiency of the human depletion steps was found to be variable, resulting in significant variation in the *RNase P* Ct values. The distribution of the Ct values for human DNA was analysed, and it was observed that optimal results were obtained when there was a lower presence of human DNA. However, the distribution of the Ct values from either MTBC or bacteria was not different between the specimens, leading to optimal or suboptimal results. It was also found that the distribution of Ct values was especially diverse in those samples corresponding to poor results. In summary, equivalent Ct values for MTBC may lead to optimal or poor results, depending on the Ct values for human or accompanying bacteria in the same specimen. Therefore, real-time PCR (RT-PCR) Ct values did not correlate with sequencing results (Kruskal–Wallis test, *X*² = 0.318, df=2, *P*=0.853).

These observations, altogether, indicate that it is challenging to find a straightforward way to predict the sequencing performance expected for a specimen.

### Allocating cases to clusters

#### Assignment from optimal sequences

In the 24 cases that demonstrated optimal results, whether from the prospective or retrospective rounds of analysis, we could determine that six of these cases (cases 3283, 3295, 3325, 3472, 3330 and 3465) corresponded to clustered cases (clusters 3133, 899-2204, 3325, 3330 and 1414), whilst the remaining cases were designated as orphan cases.

### Rescue of data from suboptimal sequences

It is acknowledged that complete genome analysis is not feasible when suboptimal results are obtained. We aimed to evaluate the potential for partial exploitation of suboptimal, at least to facilitate the pre-assignment of cases to existing clusters. The rationale behind this approach was to attempt to identify SNPs from suboptimal sequences that are markers for pre-existing clusters.

In order to address this purpose, the more epidemiologically relevant clusters in the population were first selected, namely those which were either newly identified or had grown (incorporating ≥1 case, 1–7 cases) over the last 5 years (2021–2025). A total of 34 clusters were selected (Table 2).

**Table 2.** Common, core marker and within-cluster SNPs for the epidemiologically relevant clusters selected

Cluster code	Cases before 2021	New cases (2021–**25**)	Common SNP	Core marker SNP	Within-cluster SNP
15	11	4	905	21*	18
29	7	2	871	25	25
459	8	1	790	22	5
625	8	2	859	15*	15
645	2	1	807	19*	4
680	1	2	803	44	2
778	2	1	791	14*	3
1101	0	2	799	45	3
1304	7	2	793	6*	11
1330	3	1	818	14	3
1414	0	2	829	13	0
1960	0	2	807	13	0
2433	4	1	811	24*	0
2819	0	2	839	27	0
2907	0	3	808	17	3
3068	0	2	825	17*	1
3084	0	2	801	35*	1
3201	0	4	829	28	1
3325	0	2	867	17	0
3420	0	2	857	9	0
113	22	3	875	5*	49
1589	4	2	787	50	6
210	2	1	801	14	1
3113	0	7	768	17*	1
3133	2	4	795	27	0
S-3313	0	2	827	18	0
3330	0	2	812	6	0
347	11	4	828	4*	22
558	4	5	819	8	4
630	4	2	798	23	2
76	21	4	778	5	12
789	31	6	840	14	16
833	11	1	844	22	13
899-2204	4	2	767	3	10

*For these clusters some sequences in the global SNP database were found sharing their marker SNPs. Once checked that they corresponded to sequences genomically/epidemiologically related to the corresponding clusters, the SNPs were still kept as general marker SNPs for the clusters.

Subsequently, the marker SNPs for the relevant clusters were identified, utilizing the genomic sequences obtained before, from the cases from the selected clusters, within our genomic surveillance programme [20]. Firstly, the common SNPs shared by all members in each cluster were determined (Table 2). Then, these were compared with an in-house database containing the SNPs from 109,648 sequences taken from publicly available databases, representing 121 countries from the year 1909 to the year 2024. Following this comparison, all cluster common SNPs found in the database were filtered out to obtain a list of a total of 641 key unique SNPs (3–50 core marker SNPs/cluster, only found in the members of each cluster; Tables 2 and S2). Furthermore, the list was expanded to encompass the differential SNPs identified between the cases included in each of the clusters (230 within-cluster SNPs; Tables 2 and S2). The total number of informative SNPs compiled for the 34 clusters selected was 871 (Table 2).

**Fig. 5.**
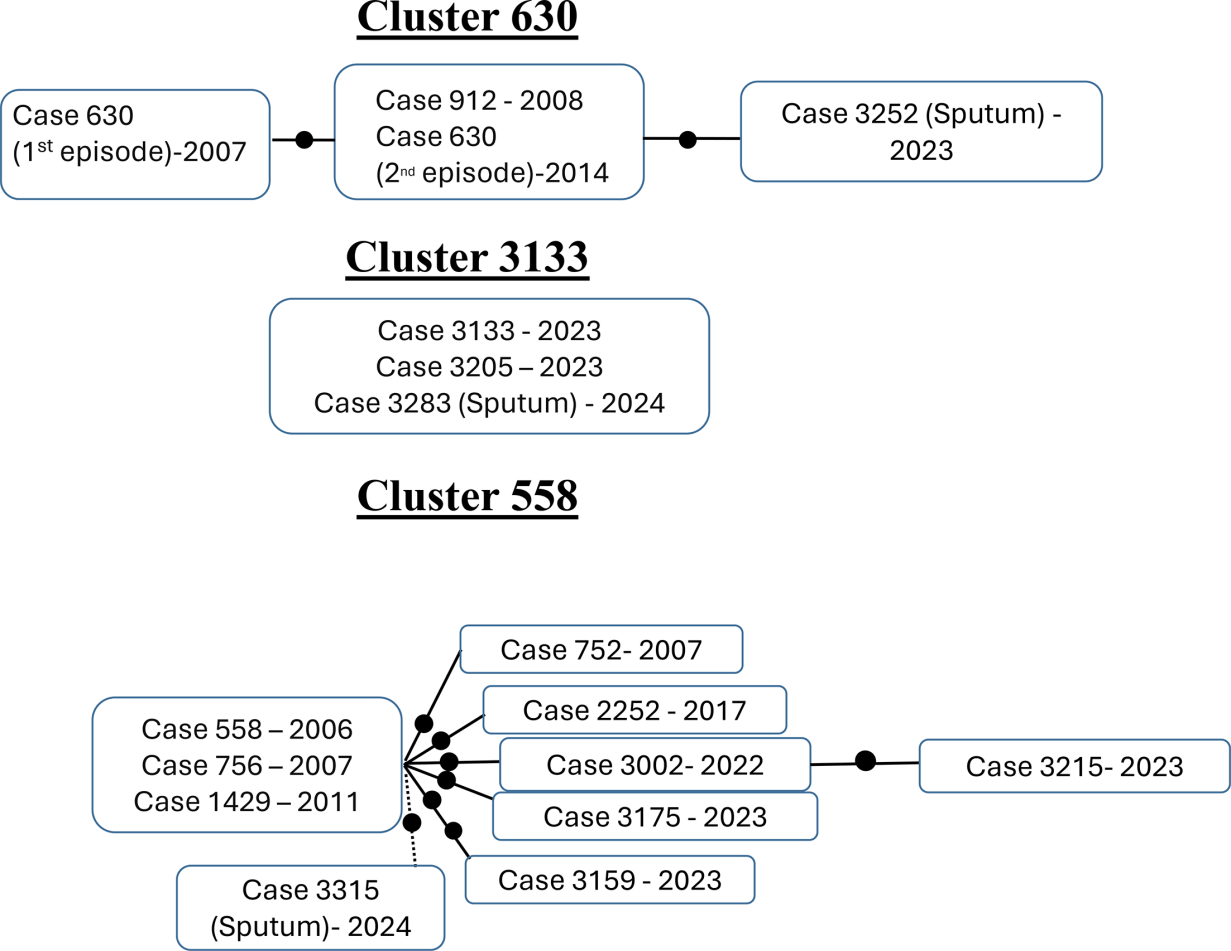
Genomic networks for the pre-existing clusters in which a new case was identified in the prospective sub-study (each dot corresponds to 1 SNP; the cases within the same box had 0 SNPs among them). Cases assigned to these clusters from sequencing analysis on sputa are indicated (sputum). In cluster 558, suboptimal sequences from case 3315 allowed us to assign it to this cluster, but the differential SNP drawn (dotted line) was obtained from the subsequent sequencing on culture.

Based on these results, a marker SNP pipeline (rescue pipeline) was customized for the purpose of calling the informative cluster SNPs from the nine suboptimal sequences that were obtained in the course of the analysis. From five of the nine cases exhibiting suboptimal sequences (1598, 755, 3315, 3252 and 3522, Table 3), a proportion of the core marker SNPs could be called, whilst no calls were obtained for the remaining four cases. Among the five samples with calls, in four of them, sufficient coverage (at ≥5×) was obtained to call 33, 2, 7 and 23 SNPs, respectively, of the corresponding cluster core marker SNPs (30%, 40%, 87.5% and 91.3% of their total cluster marker SNPs; Table 3). These calls facilitated the allocation of these subjects as probable candidates for entry into four pre-existing active clusters (clusters 1589, 113, 558 and 630). Furthermore, for patient 3252, who had been preassigned to cluster 630 by the rescue pipeline, it also enabled us to allocate it within the corresponding genomic network (Fig. 5). The correct call from the suboptimal sequences from patient 3252 of all the SNPs called in cases 630 and 912, in conjunction with one new SNP detected as acquired after analysing the suboptimal sequences, indicated that case 3252 was the last case in the network (Fig. 5). For the remaining case (case 3522), only 20% of the marker SNPs from one of the relevant clusters were called at >5× (cluster 29; Table 3), which was not considered sufficient for a pre-assignation.

**Table 3.** Samples leading to suboptimal sequences that allowed us to call cluster informative SNPs through the implementation of the rescue pipeline

Sample no.	Patient ID	Cluster	Core marker SNP	Within-cluster marker SNP	Total called_SNP	Called_Core_ SNP (%) ≥5×	Total SNP ≥ 1×	Total SNP ≥ 5×	Total SNP ≥10×
14	1598	1589	50	6	33 core	30.00	33	15	8
15	755	113	5	49	2 core	40.00	2	2	2
18	3315	558	8	4	7 core	87.50	7	4	1
23	3252	630	23	2	23 core+1 within cluster	91.30	23	21	11
71	3522	29	25	25	6 core	20.00	6	5	0

In all cases, the allocations of cases to pre-existing clusters made by the rescue pipeline from the suboptimal sequences obtained from sputa were confirmed by WGS from the corresponding cultures once they were available. This subsequent confirmatory step allowed us to ensure the accuracy of all the pre-assignments. For case 3522, in which a decision was taken not to pre-assign it to cluster 29, due to the low number of marker SNPs covered, WGS data from the culture indicated that it was indeed part of that cluster.

### Epidemiological exploitation of results from sputa

The samples included in the prospective subanalysis presented in this study enabled the evaluation of the accelerated allocation of new cases into three pre-existing clusters (cases 3252, 3283 and 3315 in clusters 630, 3133 and 558, respectively).

Cluster 630 (Fig. 5) involved two preceding migrant cases (cases 630 and 912), two siblings with TB in 2007 and 2008, respectively. Case 630 exhibited a second TB episode in 2014, consequent to inadequate adherence to anti-TB treatment during the initial episode. The inclusion of case 3252 in this cluster prompted a rapid evaluation of its relationship with the preceding cases. Indeed, as a consequence of this finding, case 3252 was interviewed, and it was revealed that he had been a close contact of case 630 during the latter’s second episode in 2014. However, he did not adhere to prophylactic treatment at that time. The rapid genomic analysis of sputum samples enabled the diagnosis of this case as a reactivation resulting from a prior exposure and, therefore, not wasting the specific control efforts that are dedicated when recent active transmissions are identified, to identify new non-diagnosed active cases.

Cluster 3133 (Fig. 5) corresponded to a recent active transmission event involving two recently arrived migrant cases (cases 3133 and 3205, diagnosed in 2023, 1.5 and 5 years after arrival, respectively) residing in a context characterized by a high proportion of the migrant population. The two preceding cases exhibited 0 SNPs between them; the inclusion of case 3283 in this cluster (also exhibiting 0 SNPs in relation to the preceding cases) indicated his involvement in the same recent common transmission event. In the absence of the rapid genomic assignment of case 3283 to this cluster, his TB would have been interpreted as a reactivation of a past exposure. This is because case 3283 had resided in Almería for 22 years prior to his diagnosis, and a type II diabetes diagnosis was made in 2024, coinciding with his diagnosis of TB.

Cluster 558 was an extensive cluster involving nine preceding cases (mainly migrants). The star-like topology of this cluster network (Fig. 5) is consistent with sequential reactivations, for most of the cases, several years after exposure to an active transmission event, which happened years before (during 2006–11). The fast allocation of case 3315 to this cluster based on genomic analysis in sputum prompted consideration of reactivation also as the most probable explanation for this case, a phenomenon that occurred in all but one of the anteceding cases in this cluster. This hypothesis was consistent with the finding of poorly controlled diabetes and the patient’s advanced age (60 years). Our preliminary consideration of reactivation based on the genomic data obtained from sputa was finally reinforced, once the sequencing data from the corresponding culture were later available. This final consolidated data allowed us to locate case 3315 in the network (Fig. 5) directly linked with the cases involved in the active transmission event which occurred in the past, also following the previously mentioned star-like topology.

## Discussion

Once WGS demonstrated the value in the identification of resistance mutations in MTB and the tracking of TB transmission with the utmost precision, efforts have been made to accelerate the availability of genomic data. The first movement was to conduct the analysis directly on primary cultures [10], obviating the necessity for subculturing. In an effort to circumvent the high-throughput schemes that cause unavoidable delays due to the necessity to accumulate a substantial number of cultures to be executed concurrently, a more flexible nanopore sequencing approach has been proposed [21]. However, in order to reduce the time lag between new diagnoses and the availability of genomic data, it is necessary to adopt a culture-free strategy by sequencing directly on sputa [22]. It is evident that this would provide the most expeditious outcomes, whilst concomitantly circumventing constraints in settings lacking access to biosafety facilities and, consequently, impeding the implementation of culture-based diagnostics.

The process of direct sequencing of MTB in sputa is a challenging task due to the presence of high amounts of accompanying DNA from either human cells or other bacteria in the respiratory specimens. This accompanying DNA can interfere with the sequencing process. In the first study, which established the feasibility of directly sequencing MTB from clinical specimens, 20–93% of the obtained sequences corresponded to human reads [6], despite the implementation of a procedure aimed at depleting human DNA present in the specimen. This finding elucidated the remarkably low coverage obtained for MTB, which even fell below the mean coverage threshold of 0.7×.

These suboptimal outcomes led to the conclusion that, when attempting to sequence directly on sputa, the enrichment in MTB provided by culturing should be substituted by alternative enrichment procedures [22]. The aforementioned enrichments have relied on indirect approaches, as in the previously mentioned article, by eliminating the interfering human DNA through differential lysis procedures [6,10, 23], or on direct schemes, by selectively capturing MTB by ligand-magnetic binding beads [3], or by hybridizing with specifically designed capture probes (biotinylated RNA baits) to selectively capture MTB libraries before sequencing [2,5, 23].

The application of an optimized procedure for the depletion of human DNA [10] resulted in enhanced coverage when conducting direct sequencing of sputa. However, the most favourable outcomes in terms of coverage depth and breadth have been achieved using RNA baits [2,5, 23], despite the fact that its application is linked to lengthier, more cumbersome and costly procedures.

To date, the scarcity of research in this field is evidenced by the paucity of studies addressing the challenge of directly sequencing MTB in sputa. This situation is particularly pertinent in low- and medium-burden settings, where further analysis is required. Moreover, the preponderance of this research has been oriented towards the evaluation of the efficiency with which resistance mutations can be identified, with a number of studies also incorporating preliminary analyses of lineage assignment or the construction of phylogenies [2,3, 5, 7]. However, the evaluation of culture-free sequencing in the context of genomic epidemiology has received minimal attention. It is evident that the acceleration of precise, personalized therapy for each patient has a discernible impact on their prognosis, thereby validating the endeavours to expedite culture-free analysis. However, in contexts such as Almeria, where epidemiological interventions are coupled and oriented by the genomic characterization of every new case [20], it is equally relevant to accelerate the genomic analysis. The timely identification and analysis of new cases is therefore paramount to orientate accordingly the effectiveness of contact tracing. This enables the enhancement of the identification and treatment of LTI cases, which in turn reduces the number of secondary cases and the overall burden of the disease. However, to the best of our knowledge, only one study [23] has addressed the culture-free genomic inference of transmission; the authors followed an innovative rationale, restricting the application of capturing-baits for the sputa with a low representativity of MTB DNA and following a direct non-capture approach for the remaining. In both branches, Illumina sequencing was employed.

The objective of our study is threefold: firstly, to address the paucity of studies dealing with culture-free sequencing of MTB in the context of genomic epidemiology; secondly, to accelerate the inference of new cases entering transmission clusters; and thirdly, to enhance our interventionist dynamic as soon as a new clustered case is identified. Looking for this acceleration, our present study proposes an alternative approach to the conventional Illumina-based sequencing method, namely, nanopore sequencing, which has the potential to enhance the analysis’s efficiency and expedite the process. A mere one of the studies on culture-free MTB sequencing is supported on nanopore sequencing [7], and it only provided rather suboptimal results because the authors decided against applying any enrichment method, in an attempt to adapt to the limited resources found in many high-burden settings. The hypothesis that nanopore is better adapted to a faster response has been demonstrated by our team in recent studies using primary cultures. This finding suggests that a more flexible case-by-case analysis of new incident cases can be performed [21]. Votintseva *et al*. also established that nanopore provided a faster response, although this finding was only supported by a pilot study using Bacillus Calmette–Guérin (BCG) spiked sputa [10].

Consequently, the present study constitutes the first analysis of culture-free sequencing of MTB with a genomic epidemiology scope supported by flexible nanopore sequencing. In this study, a depletion approach was employed; instead of a capture enrichment method, with the objective of offering a less complex and less expensive procedure. The number of specimens evaluated, 71, was the highest among other previous studies. In one-third of the cases, the quality of our results was equivalent to that obtained when sequencing directly in cultures, thus enabling us to perform a complete comparative genomics analysis. It is noteworthy that we were able to achieve substantial coverage from a 2+ specimen, and in some instances, good coverages were accomplished with short runtimes. It is important to acknowledge that in approximately half of the cases under consideration, no exploitable results, for our comparative genomics purposes, were obtained. However, it should be noted that the coverage values obtained in other studies were even below the less demanding thresholds required for tasks that are much more straightforward than comparative genomics, such as the identification of resistance mutations or the assignment of lineages [7]. Furthermore, even when employing sophisticated techniques, such as the use of magnetic beads coupled with ligands to bind and enrich MTB [3], the maximum coverages attained were only 18.7× and the overall genome coverage was 15.2%, thereby hindering even straightforward tasks, such as the assignment of lineages, which was only possible in 15% of the samples. Even some of the studies among those achieving the best results, by enriching MTB libraries by using RNA baits, faced room for improvement, with still 24% of the samples not reaching the thresholds for the purpose of comparative genomics [23].

We aimed to ascertain the underlying reasons for our proportion of unsuccessful results. Unfortunately, the comparison with sequencing data before depletion could not be obtained due to the limited volume available for the decontaminated sputa included in the study. This is also a general limitation in other studies that only provide the data obtained after depletion [10,23, 24]. We firstly evaluated the performance depending on the bacillary load, as inferred by the staining. Optimal results were only obtained in one sputum sample with a low bacillary load. All subsequent optimal results were obtained from sputum samples with higher bacillary loads. However, the performance of the sputa with higher loads ranged from poor to optimal, thus impairing predictions. Subsequently, the Ct RT-PCR values from human/other accompanying bacteria were determined. Our initial observation revealed that, despite the implementation of a differential lysis-based kit to deplete human DNA, the kit’s performance exhibited significant variability. This variability was characterized by the presence of high percentages of human DNA, or low Ct values, among the reads obtained from certain samples. The presence of human DNA even after the application of depletion procedures has also been reported in other studies [6], indicating the need for methodological improvements at this stage. The variable presence of human and bacterial DNA hindered the establishment of a criterion to predict whether a sputum sample would yield optimal or poor sequencing results. A similar absence of correlation between bacterial load or smear grade and sequencing coverage has been identified in other studies [7]. A correlation could be identified in studies that employed enrichment by RNA baits [3], thereby enabling the authors to minimize the interference of human DNA and consequently ascertain the correlation. Despite not finding a correlation between pre-analytical indicators obtained from the sample and sequencing performance, the MTB/human reads ratio obtained from the sequencing run allowed us to establish two thresholds (>45.5 or <10.93) to differentiate between optimal and poor results. We are now determining this ratio in the first 30 min of nanopore sequencing to determine whether to continue or abort the run.

It is acknowledged that obtaining WGS data directly from sputa is currently challenging [7] and that a proportion of culture-free results are expected to be suboptimal. In light of these issues, an innovative rescue pipeline was proposed by us to exploit these sequences not reaching the thresholds required for comparative genomics. The rationale behind the rescue pipeline is to identify informative SNPs in genomic regions that are adequately covered in the sequences, despite suboptimal average coverage values. The SNPs deemed informative are those which were identified to be exclusively present in the relevant clusters within our population; thus, they can function as cluster marker SNPs. Consequently, if we can call them in a set of suboptimal sequences, we can preassign the corresponding case to a pre-existing relevant cluster. The potential of marker SNPs to preassign a strain to a certain cluster has been demonstrated by our team in many preceding studies and settings. This has been achieved by using targeted PCRs to track relevant strains in France, Panama, Costa Rica, Morocco, Belgium, Peru and others in a faster, simpler and low-cost way [20,25,29]. Marker SNPs have also been proposed as a means of facilitating more efficient and expeditious identification of the primary multi-drug resistant (MDR) strains circulating in Europe [30]. The current rescue pipeline utilizes the same concepts but now seeks these marker SNPs among the calls obtained from samples with suboptimal results. This approach enabled us to identify cases of pre-existing clusters in several instances, and in one particular instance, it facilitated the precise location of the case within the genomic network of the cluster. In all these cases, the pre-assignments, supported by the identification of a subset of marker SNPs, were validated by the complete sequencing data obtained at a later stage, once the cultures were available.

It is true that the inference of clusters in a population from culture-free data, particularly in the context of implementing a rescue pipeline, necessitates the pre-existence of a long-term surveillance programme. This programme facilitates the identification of the most relevant clusters, as determined by their size and the rate at which new cases have entered those clusters. In this particular instance, the rapid identification of genomic assignments for new cases to pre-existing clusters, within the prospective subanalysis, offered special value. Indeed, the findings of this study have enabled us to ascertain when the new entrances were more likely to be the result of either reactivations due to past exposures or active recent transmission. It is recommended that special efforts to enhance transmission control should particularly focus on the cluster associated with active transmission. The new entrances in the other two clusters, attributable to the reactivation of prior exposures, underscore the challenges inherent in ensuring comprehensive coverage in contact tracing within these epidemiologically intricate populations characterized by high rates of migration and consequently in ensuring the administration of prophylactic treatment for tuberculosis infection in all pertinent cases.

In summary, the present study provides additional insights into the possibilities of culture-free WGS of MTB in the context of interventionist precision genomic epidemiology. Despite the identification of scope for enhancement, the findings of this study initiate the process of integrating a rapid analysis in close proximity to diagnosis and a preliminary categorization of cases, despite suboptimal outcomes.

## Supplementary material

10.1099/mgen.0.001709 Uncited Table S1.

10.1099/mgen.0.001709 Uncited Table S2.

10.1099/mgen.0.001709 Uncited Table S3.

## Abbreviations

MTB: *Mycobacterium tuberculosis*
TBPCP: TB Prevention and Control Program
WGS: whole-genome sequencing
