# Supplementary material for: Direct nanopore sequencing of Mycobacterium tuberculosis on sputa and rescue of suboptimal results to enhance transmission surveillance
Source: Microb Genom. 2026 May 22;12(5):001709. doi: 10.1099/mgen.0.001709 (PMC13196888; doi:10.1099/mgen.0.001709)
Supplement: Uncited Table S1. [file mgen-12-01709-s001.pdf]

Supplementary table 1: Genome coverages of 71 sputa ≥10X, 20X and 30X along with the flow-cell version used for each run.

Patient ID	Accession number	Sample	Genome coverage ≥ 10X	Genome coverage ≥ 20X	Genome coverage ≥ 30X	Flow-cell version
33490676-2	ERS26934155	1	0,25	0,03	0	
3255	ERS26934156	2	90,92	49,06	11,69	R09
3257	ERS26934157	3	0	0	0	
3268	ERS26934158	4	0	0	0	
3262	ERS26934159	5	0	0	0	R10
3265	ERS26934160	6	0	0	0	
3281	ERS26934161	7	0,04	0,02	0	R10
3290	ERS26934162	8	0,12	0,1	0,1	
1597	ERS26934163	9	1,49	0,72	0.51	R09
1165	ERS26934164	10	99,49	99,25	99,06	
3278	ERS26934165	11	47,19	37,19	29.63	R09
3280	ERS26934166	12	10,33	3,72	1.49	
691	ERS26934167	13	97,27	96,09	93,57	R09
1598	ERS26934168	14	25,28	13,61	8.46	
755	ERS26934169	15	27,57	19,76	15.66	R09
3299	ERS26934170	16	98,73	98,37	98,06	
3313	ERS26934171	17	1,43	0,7	0,4	
3315	ERS26934172	18	15,2	3,78	1,14	R10
3296	ERS26934173	19	98,89	98,52	98,24	
3283	ERS26934174	20	97,51	96,08	94.29	
3292	ERS26934175	21	0,01	0	0	R10
630	ERS26934176	22	0,76	0,32	0,16	
3252	ERS26934177	23	27,96	3,37	0,69	
3302	ERS26934178	24	98.09	96.96	95,91	
3319	ERS26934179	25	99.01	98.8	98,69	R10
3295	ERS26934180	26	95.06	88.42	80,2	
3309	ERS26934181	27	97.39	95.9	96,95	R10
3325	ERS26934182	28	97.89	96.78	96,18	
3324	ERS26934183	29	3,18	2,78	2,25	
3330	ERS26934184	30	98.66	98.06	97,65	R10
3333	ERS26934185	31	80,28	74,56	69,25	
3334	ERS26934186	32	92,17	88,06	84,01	
3344	ERS26934187	33	98.33	97.36	96,49	
3345	ERS26934188	34	99.72	99.68	99,55	R10
3339-33272407	ERS26934189	35	3.4	2.49	2,13	
3339-14216402	ERS26934190	36	0.07	0.04	0,01	

<table>
<tr><td>3364</td><td>ERS26934191</td><td>37</td><td>99,12</td><td>98,4</td><td>97,63</td><td rowspan="3">R10</td></tr>
<tr><td>3366</td><td>ERS26934192</td><td>38</td><td>0</td><td>0</td><td>0</td></tr>
<tr><td>3367</td><td>ERS26934193</td><td>39</td><td>0,12</td><td>0,11</td><td>0,1</td></tr>
<tr><td>3370</td><td>ERS26934194</td><td>40</td><td>5,11</td><td>2,89</td><td>1,9</td><td>R10</td></tr>
<tr><td>1510</td><td>ERS26934195</td><td>41</td><td>15.55</td><td>5.96</td><td>3,16</td><td rowspan="2">R10</td></tr>
<tr><td>1876</td><td>ERS26934196</td><td>42</td><td>48.99</td><td>27.97</td><td>17,45</td></tr>
<tr><td>1925</td><td>ERS26934197</td><td>43</td><td>1,22</td><td>0,89</td><td>0,75</td><td rowspan="2">R10</td></tr>
<tr><td>2024</td><td>ERS26934198</td><td>44</td><td>1,65</td><td>0,23</td><td>0,02</td></tr>
<tr><td>3373</td><td>ERS26934199</td><td>45</td><td>0.1</td><td>0.1</td><td>0,07</td><td rowspan="4">R10</td></tr>
<tr><td>3378</td><td>ERS26934200</td><td>46</td><td>1.11</td><td>0.31</td><td>0,16</td></tr>
<tr><td>3379</td><td>ERS26934201</td><td>47</td><td>0.09</td><td>0.02</td><td>0</td></tr>
<tr><td>3382</td><td>ERS26934202</td><td>48</td><td>0.03</td><td>0.0</td><td>0</td></tr>
<tr><td>3388</td><td>ERS26934203</td><td>49</td><td>0,8</td><td>0,39</td><td>0,16</td><td>R10</td></tr>
<tr><td>3389</td><td>ERS26934204</td><td>50</td><td>5,64</td><td>0,98</td><td>0,31</td><td rowspan="2">R10</td></tr>
<tr><td>3393</td><td>ERS26934205</td><td>51</td><td>0,23</td><td>0,04</td><td>0,01</td></tr>
<tr><td>3417</td><td>ERS26934206</td><td>52</td><td>1,81</td><td>0,08</td><td>0</td><td rowspan="4">R10</td></tr>
<tr><td>3420</td><td>ERS26934207</td><td>53</td><td>4,27</td><td>4,27</td><td>2,92</td></tr>
<tr><td>3429</td><td>ERS26934208</td><td>54</td><td>95,41</td><td>82,64</td><td>49,82</td></tr>
<tr><td>3419</td><td>ERS26934209</td><td>55</td><td>0</td><td>0</td><td>0</td></tr>
<tr><td>3450</td><td>ERS26934210</td><td>56</td><td>99,05</td><td>98,58</td><td>98,12</td><td rowspan="4">R10</td></tr>
<tr><td>3455</td><td>ERS26934211</td><td>57</td><td>3,18</td><td>2,34</td><td>1,85</td></tr>
<tr><td>3465</td><td>ERS26934212</td><td>58</td><td>97,16</td><td>95,98</td><td>94,85</td></tr>
<tr><td>3467</td><td>ERS26934213</td><td>59</td><td>1,27</td><td>0,4</td><td>0,2</td></tr>
<tr><td>3474</td><td>ERS26934214</td><td>60</td><td>12,02</td><td>2,93</td><td>1</td><td rowspan="4">R10</td></tr>
<tr><td>3479</td><td>ERS26934215</td><td>61</td><td>0</td><td>0</td><td>0</td></tr>
<tr><td>3481</td><td>ERS26934216</td><td>62</td><td>0,33</td><td>0,09</td><td>0,05</td></tr>
<tr><td>3472</td><td>ERS26934217</td><td>63</td><td>96,22</td><td>86,77</td><td>62,78</td></tr>
<tr><td>3462</td><td>ERS26934218</td><td>64</td><td>94,73</td><td>90,76</td><td>85,96</td><td rowspan="4">R10</td></tr>
<tr><td>3488</td><td>ERS26934219</td><td>65</td><td>98,65</td><td>98,32</td><td>97,72</td></tr>
<tr><td>3500</td><td>ERS26934220</td><td>66</td><td>10,55</td><td>4,75</td><td>2,49</td></tr>
<tr><td>3502</td><td>ERS26934221</td><td>67</td><td>0</td><td>0</td><td>0</td></tr>
<tr><td>3503</td><td>ERS26934222</td><td>68</td><td>97,63</td><td>96,46</td><td>95,03</td><td rowspan="4">R10</td></tr>
<tr><td>3524</td><td>ERS26934223</td><td>69</td><td>0,03</td><td>0,03</td><td>0</td></tr>
<tr><td>3523</td><td>ERS26934224</td><td>70</td><td>96,92</td><td>95,4</td><td>93,21</td></tr>
<tr><td>3522</td><td>ERS26934225</td><td>71</td><td>73,9</td><td>66,27</td><td>59,49</td></tr>
</table>
