# Supplementary material for: Direct nanopore sequencing of Mycobacterium tuberculosis on sputa and rescue of suboptimal results to enhance transmission surveillance
Source: Microb Genom. 2026 May 22;12(5):001709. doi: 10.1099/mgen.0.001709 (PMC13196888; doi:10.1099/mgen.0.001709)
Supplement: Uncited Table S3. [file mgen-12-01709-s003.pdf]

Supplementary table 3

Cultured isolate	Accession number	Cluster
3252	ERS29556356	630
3283	ERS29556357	3133
3315	ERS29556358	3315
